# Impact of the COVID-19 Pandemic on Hepatitis C Treatment Initiation in British Columbia, Canada: An Interrupted Time Series Study

**DOI:** 10.3390/v16050655

**Published:** 2024-04-23

**Authors:** Richard L. Morrow, Mawuena Binka, Julia Li, Mike Irvine, Sofia R. Bartlett, Stanley Wong, Dahn Jeong, Jean Damascene Makuza, Jason Wong, Amanda Yu, Mel Krajden, Naveed Zafar Janjua

**Affiliations:** 1 British Columbia Centre for Disease Control, Vancouver, BC V5Z 4R4, Canada; richard.morrow@ubc.ca (R.L.M.); julia.li@bccdc.ca (J.L.); mike.irvine@bccdc.ca (M.I.); sofia.bartlett@bccdc.ca (S.R.B.); stanley.wong@bccdc.ca (S.W.); dahn.jeong@bccdc.ca (D.J.); jean.makuza@bccdc.ca (J.D.M.); jason.wong@bccdc.ca (J.W.); amanda.yu@bccdc.ca (A.Y.); mel.krajden@bccdc.ca (M.K.); 2 School of Population and Public Health, University of British Columbia, Vancouver, BC V6T 1Z3, Canada; 3 Faculty of Health Sciences, Simon Fraser University, Burnaby, BC V5A 1S6, Canada; 4 Department of Pathology and Laboratory Medicine, University of British Columbia, Vancouver, BC V6T 1Z4, Canada; 5 Centre for Health Evaluation and Outcome Sciences, St Paul’s Hospital, Vancouver, BC V6Z 1Y6, Canada

**Keywords:** British Columbia, cascade, COVID-19, hepatitis C, interrupted time series analysis

## Abstract

We investigated the impacts of the COVID-19 pandemic on hepatitis C (HCV) treatment initiation, including by birth cohort and injection drug use status, in British Columbia (BC), Canada. Using population data from the BC COVID-19 Cohort, we conducted interrupted time series analyses, estimating changes in HCV treatment initiation following the introduction of pandemic-related policies in March 2020. The study included a pre-policy period (April 2018 to March 2020) and three follow-up periods (April to December 2020, January to December 2021, and January to December 2022). The level of HCV treatment initiation decreased by 26% in April 2020 (rate ratio 0.74, 95% confidence interval [CI] 0.60 to 0.91). Overall, no statistically significant difference in HCV treatment initiation occurred over the 2020 and 2021 post-policy periods, and an increase of 34.4% (95% CI 0.6 to 75.8) occurred in 2022 (equating to 321 additional people initiating treatment), relative to expectation. Decreases in HCV treatment initiation occurred in 2020 for people born between 1965 and 1974 (25.5%) and people who inject drugs (24.5%), relative to expectation. In summary, the pandemic was associated with short-term disruptions in HCV treatment initiation in BC, which were greater for people born 1965 to 1974 and people who inject drugs.

## 1. Introduction

Chronic hepatitis C virus (HCV) infection affects approximately 58 million people worldwide and may result in serious morbidity and death if untreated [1]. In 2016, Canada joined the World Health Organization (WHO) campaign to eliminate HCV as a public health threat, through an 80% reduction in the incidence of chronic HCV infections, increases to a 90% coverage of diagnosis and an 80% coverage of treatment, and a 65% reduction in related mortality by 2030 [2,3]. While direct-acting antivirals (DAAs) represent a highly effective treatment that can cure chronic HCV infection in most cases [4], the COVID-19 pandemic has added to the challenges [5] faced by Canada and other countries in meeting targets for diagnosis and treatment.

A growing number of studies are helping to characterize the impact of the COVID-19 pandemic and related policies on the HCV care cascade [6,7,8,9,10,11,12]. In British Columbia (BC), Canada, HCV testing decreased immediately following the introduction of pandemic-related policies in March 2020, but recovered to near pre-pandemic levels by the end of 2020 [6]. A survey of European and non-European clinical centres suggested that the pandemic was associated with reductions in consultations, new referrals, HCV ribonucleic acid (RNA) detection, and new antiviral treatments during 2020 [7]. The pandemic was associated with reductions in HCV testing during April 2020 to May 2021 in Ontario, Canada [8].

An international study of HCV treatment suggested 46 of 54 jurisdictions studied (including countries and regions) experienced a decrease in DAA utilization during the first 6 months of the pandemic (March to August 2020), showing an average reduction of 43% compared to the same period in 2019 [13]. Among G7 countries included in the same study, statistically significant reductions in DAA utilization during March to August 2020 were found in Canada (−18%), Germany (−19%), the United Kingdom (−32%), and the United States (−24%). A study using national data from April 2018 to May 2021 suggested that DAA utilization in the United States (US) gradually increased following an abrupt decrease in the spring of 2020 but did not recover to pre-pandemic levels by April 2021 [14].

While Canada and many other countries experienced decreases in DAA utilization early in the COVID-19 pandemic, the pandemic’s longer-term impact on HCV treatment and its impact on HCV treatment in specific population groups are unclear [13,14,15,16]. Most previous studies were conducted using aggregated dispensation data, limiting their ability to assess impacts among specific populations most affected by HCV, including people who inject drugs (PWID). PWID were also disproportionately affected by COVID-19 [17,18,19,20]. We conducted an interrupted time series analysis to investigate potential changes in HCV treatment initiation following the introduction of pandemic-related policies, including impacts from April 2020 to December 2022 and impacts in the full BC population by sex, birth cohort, and injection drug use status.

## 2. Materials and Methods

### 2.1. Data Sources

We used linked administrative health data assembled in the BC COVID-19 Cohort, including data on medication dispensations (BC PharmaNet), physician billings (BC Medical Services Plan), hospital admissions (Discharge Abstracts Database), and emergency department visits (National Ambulatory Care Reporting System) [21,22,23,24,25,26,27,28,29,30,31,32,33,34,35,36] (Supplementary Materials Table S1). Medication data comprised dispensations from all community pharmacies in the province, including records for most of the BC population. Medication dispensing records were unavailable for beneficiaries of the BC First Nations Health Authority Health Benefits Program or members of federal public drug benefit programs (eligible members of the Canadian Armed Forces, qualified veterans by Veterans Affairs Canada, members of the Royal Canadian Mounted Police, and people incarcerated in federal correctional facilities) [37,38]. As of 1 April 2020, the BC population was estimated to be 5.2 million.

### 2.2. Study Population and Design

The study included BC residents who initiated HCV treatment during our study period of April 2018 to December 2022. The study period included a 24-month pre-policy period (April 2018 to March 2020) and three post-policy periods, including March to December 2020, January to December 2021, and January to December 2022.

Public reimbursement of DAAs in BC was expanded in mid-March 2018 to all individuals with HCV, regardless of the severity of their disease [39]. Preliminary analysis of data prior to March 2020 to inform our study design suggested this coverage change was associated with a peak in HCV treatment initiation in May to June 2018 and a subsequent decline (Figure S1). We chose to use a pre-policy period of April 2018 to March 2020 in our study because this allowed us to model this declining trend, in addition to possible seasonality and autocorrelation prior to anticipated effects of the pandemic and pandemic-related policies on HCV treatment initiation.

In mid-March 2020, public officials in BC declared a public health state of emergency (17 March) and provincial state of emergency (18 March) [40]. These declarations ushered in initial pandemic-related policies such as school closures, a suspension of in-person dining in restaurants, a recommendation to adopt work-from-home policies, and temporary closure of non-essential health services [40]. While restrictions on non-essential health care services were eased on 18 May 2020, pandemic-related restrictions on movement continued in varying forms during and after 2020 [40]. As the emergency declarations in March 2020 represented a major shift of policy and also likely coincided with a change in the public’s perceptions of the pandemic, we designed our study to model a pre-policy phase ending in March 2020 and post-policy phase from April 2020 to December 2022. We further designated three post-policy periods within the post-policy phase to capture evolution of changes in HCV treatment initiation following the major shift in policy in March 2020.

We used interrupted time series analyses [41] with monthly measures of HCV treatment initiation to evaluate the potential impact of the pandemic and related policy measures in each of the three post-policy periods. We conducted analyses of the full population of BC and analyses stratified by sex, birth cohort (born before 1945, 1945 to 1964, 1965 to 1974, and 1975 or later), and injection drug use status. Injection drug use was defined by two physician visits, one hospitalization or one emergency department visit with a diagnostic code related to misuse of an injectable drug, occurring prior to the end of the study period and while the individual was between 11 and 65 years of age, inclusive (see Supplementary Materials for detailed definition). A similar definition, which did not include emergency department visits, was estimated to have 78% specificity and 83% sensitivity [42].

### 2.3. Statistical Analysis

The study outcome was the monthly number of individuals who initiated HCV treatment, where initiation was defined as a first dispensation of HCV medication since the start of 2015 (see Table S2 in the Supplementary Materials for a list of relevant HCV medications). After our preliminary analysis of pre-policy data identified a non-linear trend in HCV treatment initiation, we chose to adopt an approach to interrupted time series analysis with generalized additive models (GAMs), which has been demonstrated to perform well in modelling post-policy changes in outcomes in the presence of non-linearity [43]. The statistical models used a log link function with a negative binomial distribution, which was chosen because it was appropriate for modelling count data and preliminary analyses, assuming a Poisson distribution showed overdispersion. Models included a binary variable to evaluate a post-policy level change, a smoothing function for the pre-policy trend, a smoothing function to adjust for seasonality, and a smoothing function for the post-policy trend [43]. Based on a review of autocorrelation and partial autocorrelation plots, we also adjusted our analyses for autocorrelation.

Our statistical approach allowed us to estimate a rate ratio representing the post-policy change in the level of HCV treatment initiation, reflecting an abrupt shift in HCV treatment initiation occurring at the outset of the post-policy period. It also allowed us to calculate the absolute and percentage difference in post-policy HCV treatment initiation over each of the three post-policy periods due to the pandemic and pandemic-related policies, by taking the difference between model estimates of HCV treatment initiation based on pre- and post-policy data and estimated counterfactuals based on pre-policy data only.

Interrupted time series regression analyses with general additive models were conducted using the package mgcv in R [44]. Ethical approval was obtained from the University of British Columbia Behavioural Research Ethics Board (H20-02097). Informed consent was not required, because the study used de-identified, secondary data sources.

## 3. Results

During the pre-policy period (April 2018 to March 2020), the median monthly number of individuals initiating HCV treatment in the full BC population was 256 (interquartile range 89.5) (Table S3). The median monthly number of individuals initiating HCV treatment during this period was higher among males compared to females, higher among those born from 1945 to 1964 (“baby boomers”) compared to other birth cohorts, and higher among people who inject drugs compared to those who do not inject drugs (Table S3).

**Figure 1:**
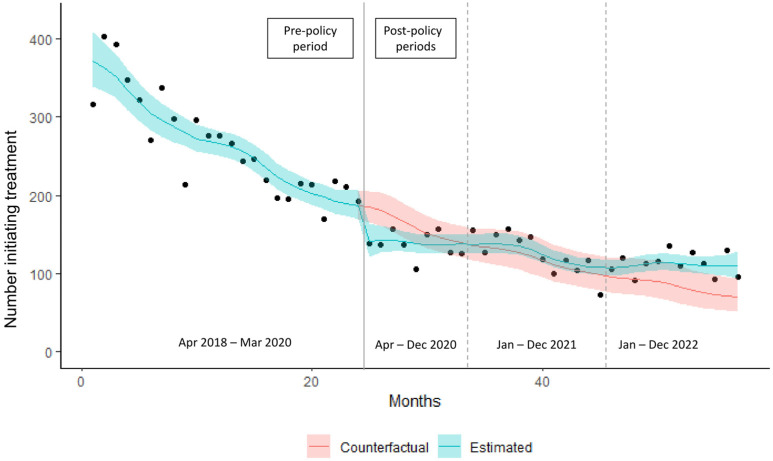
Estimated number of individuals initiating hepatitis C treatment following COVID-19-related policies in British Columbia with 95% confidence band, compared to counterfactual, April 2018–December 2022.

HCV treatment initiation followed a declining trend during the pre-policy period. At the beginning of the post-policy phase in April 2020, there was an abrupt decrease in HCV treatment initiation (Figure 1). This abrupt decrease in HCV treatment initiation in April 2020 was reflected in a statistically significant level change indicating a 26% (rate ratio 0.74, 95% CI 0.60 to 0.91) reduction in treatment initiation (Table 1). This was followed by a weakening of the downward trend in HCV treatment initiation, and there was no statistically significant difference in estimated HCV treatment initiation in the 2020 and 2021 post-policy periods. There was an increase of 34.4% in HCV treatment initiation in 2022 (equating to 321 additional people who initiated treatment), relative to the counterfactual estimates (or “expectation”) (Table 2, Figure 1). Despite this increase above the counterfactual trend in 2022, HCV treatment initiation was 52.3% lower in 2022 compared to 2019, due to declining pre- and post-policy trends in HCV treatment initiation (Table S4).

The stratified analyses suggested that changes in HCV treatment initiation following the introduction of pandemic-related policies differed by sex, birth cohort, and injection drug use status. Female and male populations each experienced a negative shift in the level of HCV treatment initiation of about one-fifth in the post-policy phase (Table 1, Figure S2), and neither of these populations experienced a statistically significant difference in HCV treatment initiation over the 2020 and 2021 post-policy periods (Table 2). However, HCV treatment initiation increased relative to expectation among females but not males in 2022 (Table 2).

**Figure 2:**
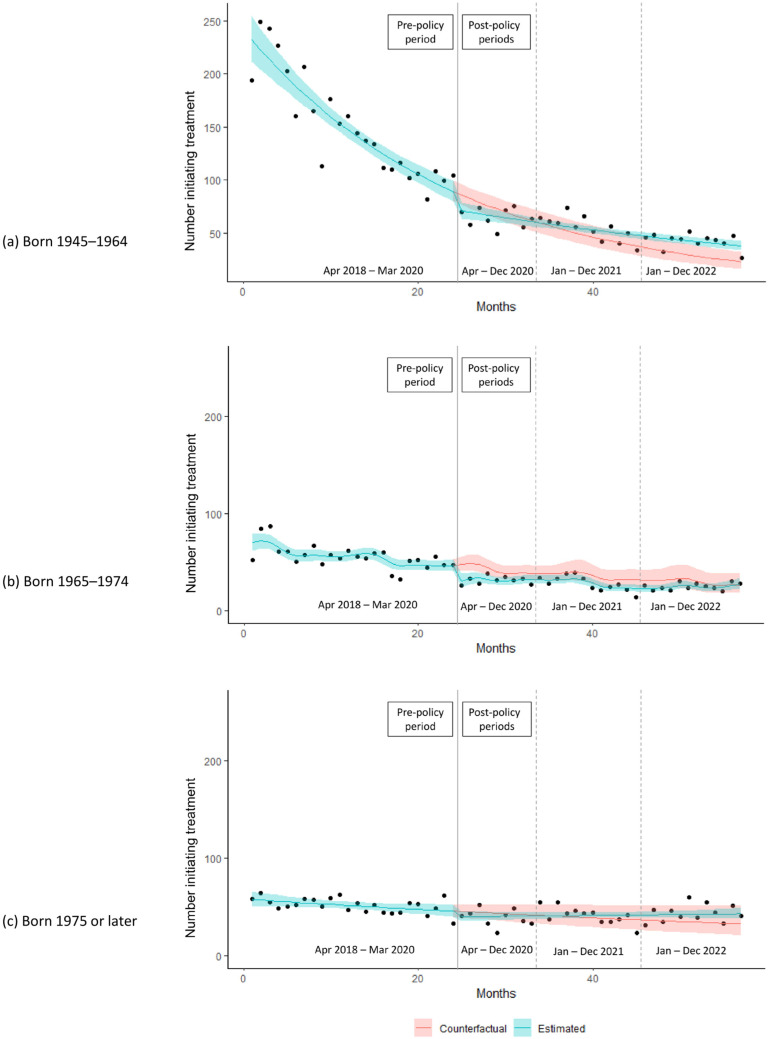
Estimated number of individuals initiating hepatitis C treatment following COVID-19-related policies with 95% confidence band, compared to counterfactual, stratified by birth cohort. The cohort of individuals born prior to 1945 is omitted due to small numbers.

Individuals born between 1965 and 1974 experienced an abrupt decrease in the level of HCV treatment initiation of 40% in April 2020, which was double the 19% decrease among baby boomers, while other birth cohorts showed no statistically significant decrease in level, relative to expectation (Table 1). Those born between 1965 and 1974 also experienced a statistically significant decrease in HCV treatment initiation during the 2020 post-policy period of 25.5%, compared to no significant decrease in other birth cohorts (relative to expectation). In contrast, baby boomers were the only birth cohort to show a statistically significant increase in HCV treatment initiation in 2022, with an increase of 48.4%, compared to expectation (Table 2, Figure 2).

**Figure 3:**
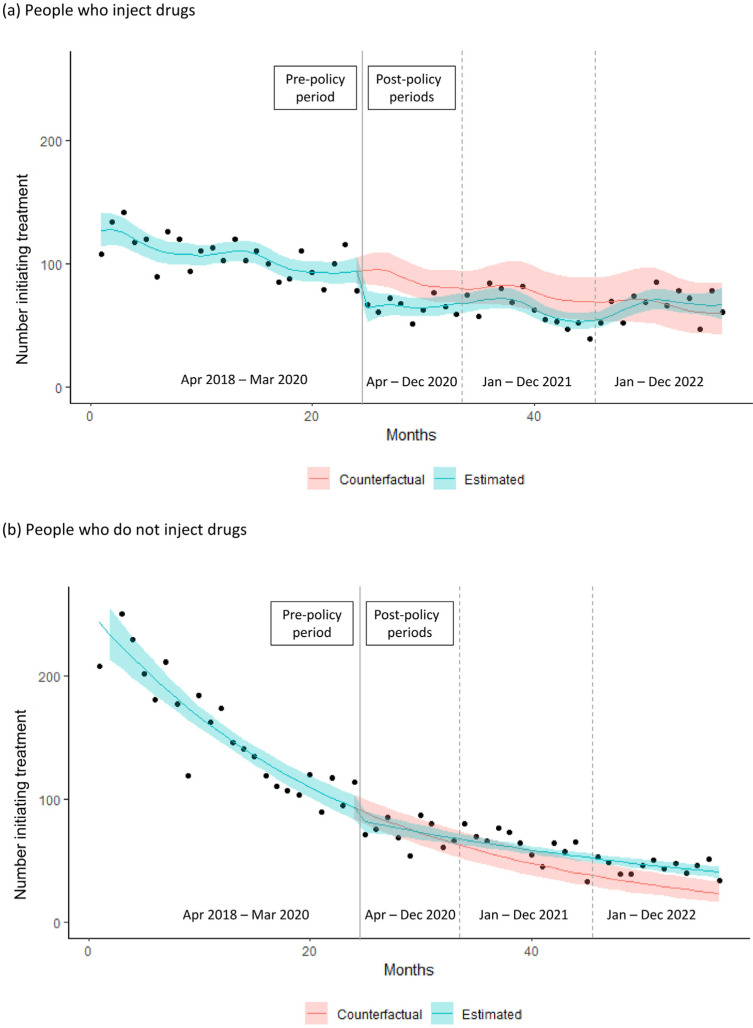
Estimated number of individuals initiating hepatitis C treatment following COVID-19-related policies with 95% confidence band, compared to counterfactual, stratified by injection drug use status.

People who inject drugs experienced a statistically significant decrease in the level of HCV treatment initiation of one-third in April 2020, compared to a non-significant decrease of 11% among those who do not inject drugs (Table 1). Among people who inject drugs, HCV treatment initiation decreased by 24.5% in the 2020 post-policy period relative to expectation, while there was no statistically significant change among those who do not inject drugs during the same period (Table 2, Figure 3). In contrast, among people who do not inject drugs, HCV treatment initiation increased by 55.4% in 2022, while there was no statistically significant change in HCV treatment initiation among people who inject drugs, relative to expectation.

## 4. Discussion

In this population-based cohort, we showed that the COVID-19 pandemic and related policies were associated with a decrease in HCV treatment initiation in BC, but this effect was temporary; by 2022, we observed that HCV treatment initiation exceeded expected treatment initiation (counterfactual estimates derived from pre-policy trends). Changes in HCV treatment initiation following the introduction of pandemic-related policies varied by sex, birth cohort, and injection drug use status. The female and male populations experienced a similar immediate decrease in the level of HCV treatment initiation in April 2020, but HCV treatment initiation rose above expectation in 2022 only among females. Among birth cohorts, only people born between 1965 and 1974 experienced a decrease in HCV treatment initiation across the 2020 post-policy period, relative to expectation, and only people born between 1945 and 1964 experienced an increase in HCV treatment initiation in 2022 compared to expectation. Similarly, people who inject drugs experienced a decrease in HCV treatment initiation across the 2020 post-policy period relative to expectation, while HCV treatment initiation among people who do not inject drugs did not differ from expectation over the 2020 post-policy period but exceeded expectation in 2022. Notably, HCV treatment initiation was not lower than counterfactual estimates in any population group in 2022, but the total number of individuals who initiated HCV treatment was approximately 50% lower in 2022 compared to 2019, due to declining pre- and post-policy trends. These analyses show that a recovery from the decline in HCV treatment initiation related to the pandemic occurred, though overall treatment initiation was lower than during 2019. Additional interventions will be needed to scale up testing and treatment initiatives, in order to achieve HCV elimination targets.

Patterns of HCV treatment initiation in BC from early in the COVID-19 pandemic until the end of 2022 were likely related to a range of factors, including pandemic-related policies, barriers to accessing health services, and the public’s response to the pandemic. Early in the pandemic, non-essential health services (23 March to 19 May 2020) and non-urgent HCV testing (1 April to 28 May 2020) were temporarily suspended in BC [6,40]. Along with other public policies, these measures likely had an impact on both HCV testing [6] and HCV treatment initiation early in the spring of 2020. Conversely, the gradual recovery of HCV testing rates during the course of 2020 likely helped with the recovery of the trend in monthly HCV treatment initiation. Our findings suggest that the decrease in HCV treatment initiation during the period from April to December 2020 primarily affected people who inject drugs; this likely stems from greater barriers in access to health care services among individuals in this group, due to factors such as the transition to greater use of telemedicine in primary care and increased requirements to schedule appointments for lab testing. If males were more likely to inject drugs than females, as may be suggested by the consistently higher number of “illicit drug toxicity deaths” among males compared to females reported by the BC Coroners Service [45], this may help explain our finding that HCV treatment initiation rose above expectation for females but not males in 2022.

The declining pre- and post-policy trends in HCV treatment initiation in BC may be related to various factors. The expansion of public reimbursement of DAAs in BC [39] in mid-March 2018 to all individuals, regardless of the severity of their disease, was likely related to the treatment of people waiting for an expansion in treatment eligibility. This resulted in an increase in HCV treatment, which peaked in May to June 2018 with a subsequent decline, due to a declining number of people diagnosed and engaged with care. Our study illustrates that the COVID-19 pandemic temporarily exacerbated this declining trend in HCV treatment initiation. The reasons that HCV treatment initiation rose above expectation in 2022 among people who do not inject drugs but not among people who inject drugs are unclear. However, HCV treatment initiation among people who inject drugs might have been hampered by the ongoing public health emergency of unregulated drug poisoning (overdose) in the province [45]. Overall, efforts will be needed to increase the linkage with the care of those already diagnosed and the diagnosis of those who remain undiagnosed and link them with care. These efforts will require tailoring interventions for various population groups, such as PWID.

Our study findings are consistent with other studies that have suggested HCV treatment declined early in the COVID-19 pandemic, including in Canada, the US, and in many other countries around the world [13,14,15,16]. We found that the level of HCV treatment initiation decreased immediately following the introduction of pandemic-related policies, although our findings did not indicate that HCV treatment initiation differed from expectation over the period from April to December 2020, except in certain populations. Our findings contrasted with a study that suggested DAA utilization in the US remained lower than expected until the end of 2020 [15].

The COVID-19 pandemic may have affected the HCV infection and disease burden in BC. While HCV transmission may have increased among people who inject drugs due to disruption in access to harm reduction services [2], the reduction in HCV treatment initiation found in our study might also have weakened the potential for treatment to act as prevention, particularly among people who inject drugs [46,47]. Among birth cohorts, people born between 1965 and 1974 were shown to account for the second highest number of infections in BC (after those born 1945 to 1964) [48], and our study shows HCV treatment initiation was more affected by the pandemic for this birth cohort compared to others. The impact of pandemic on HCV-related outcomes overall and in various population groups is not known. Studies are needed to characterize the impact of pandemic on HCV-related outcomes.

The new contributions of this study include an investigation into possible longer-term effects of the pandemic on HCV treatment initiation and differential impacts on specific populations. Earlier studies on the impact of the pandemic on HCV treatment examined impacts during 2021 and early 2021 [13,14,15,16]. Our finding, that HCV treatment initiation was not lower than expected in 2021 or 2022, suggests that the impact of the pandemic on HCV treatment initiation dissipated over time in BC. Previous studies have not been able to investigate impacts on specific populations most affected by HCV, due to data limitations, such as the use of aggregate data lacking person-level characteristics [13,14,15,16]. Our study indicates that the pandemic had a greater impact during April to December 2020 among people born from 1965 to 1974 and people who use injection drugs.

A modelling study suggested that targets related to the WHO campaign to eliminate HCV as a public health threat could be met by 2028 in BC, based on the assumptions of annual diagnosis at the 2017–2018 level and treatment at the 2019 level [2]. The study further estimated that HCV elimination would be delayed to 2040, if there were a 10% reduction in diagnosis and treatment from assumed values. Our findings indicate that HCV treatment initiation was only disrupted temporarily by the COVID-19 pandemic, but that the level of HCV treatment initiation was approximately 50% lower in 2022 compared to 2019, due to declining trends in HCV treatment initiation before and after the start of the pandemic. This decline in HCV treatment initiation threatens to delay the fulfilment of HCV elimination goals in BC beyond 2030, but the decline started prior to the pandemic and the pandemic has represented only one among other challenges in meeting these goals. The pre-pandemic decline in HCV treatment initiation was related to a decline in the number of people who were already diagnosed, engaged in care, and waiting for DAA treatments. An increase in HCV testing will be needed to diagnose people living with HCV and address the decline in HCV treatment initiation. Our study also highlights the challenge of providing HCV care to people who inject drugs. Thus, for achieving HCV elimination targets, there is a need to scale up testing and treatment overall and specifically among population groups which are disproportionately affected by HCV [49] and faced greater challenges during the pandemic, such as people who inject drugs.

The strengths of our study include the use of a rigorous interrupted time series design, which enabled us to account for the pre-existing trend of HCV treatment initiation even in the presence of non-linearity, and the use of linked data covering most of the BC population. We also included stratified analyses to examine HCV treatment initiation in specific populations, including two groups identified as priority populations in Blueprint to inform hepatitis C elimination efforts in Canada: people born between 1945 and 1974 (who account up to three-quarters of HCV infections) and people who inject drugs (who account for 85% of new HCV infections) [3]. However, our study was subject to limitations. We used pre-policy data to estimate counterfactuals for HCV treatment initiation for multiple periods, but counterfactuals for the period from January to December 2022 might be less reliable because this period was further from the pre-policy period. When we classified patients by injection drug use status for stratified analysis, it is likely some misclassification occurred, although this was mitigated by using a modified version of a validated algorithm [42]. We were unable to include analyses of other priority populations, namely, Indigenous peoples, people with experience in federal or provincial prisons, immigrants and newcomers from countries where HCV is common, and gay, bisexual, and other men who have sex with men [3].

## 5. Conclusions

The COVID-19 pandemic and related policies were associated with a short-term disruption in HCV treatment initiation in BC, which was greater among people born from 1965 to 1974 and people who inject drugs. This disruption in HCV treatment initiation did not continue into 2021 or 2022, but HCV treatment initiation was lower in these years compared to 2019, due to declining pre- and post-policy trends. Meeting HCV elimination targets by 2030 in BC will require additional efforts to boost HCV treatment initiation, particularly among people born from 1965 to 1974 and people who inject drugs.

## Figures and Tables

**Table 1:** Level change in the number of individuals initiating hepatitis C treatment following COVID-19-related policies in British Columbia, compared to counterfactual, by population.

Population	Rate Ratio (95% Confidence Interval)
(a) Overall	0.74 (0.60, 0.91)
(b) Sex	
Female	0.77 (0.64, 0.92)
Male	0.81 (0.71, 0.93)
(c) Birth cohort	
Before 1945	0.70 (0.37, 1.32)
1945–1964	0.81 (0.69, 0.94)
1965–1974	0.60 (0.45, 0.82)
1975 or later	0.87 (0.72, 1.05)
(d) Injection drug use status	
People who inject drugs	0.66 (0.50, 0.88)
People who do not inject drugs	0.89 (0.77, 1.04)

**Table 2:** Difference in number of individuals initiating hepatitis C treatment following COVID-19-related policies in British Columbia, compared to counterfactual, by population and period.

Population	Absolute Difference, n (95% Confidence Interval)			Percentage Difference, % (95% Confidence Interval)		
	April–December 2020	January–December 2021	January–December 2022	April–December 2020	January–December 2021	January–December 2022
(a) Overall	−191.0 (−401.4, 8.4)	77.8 (−227.1, 348.5)	321.3 (9.7, 585.5)	−12.9 (−25.0, 0.6)	6.4 (−13.3, 29.3)	34.4 (0.6, 75.8)
(b) Sex						
Female	−56.8 (−147.2, 26.7)	70.1 (−52.8, 174.1)	174.2 (55.3, 277.5)	−11.8 (−27.8, 6.7)	18.5 (−9.7, 52.3)	67.3 (13.3, 137.9)
Male	−126.5 (−274.5, 13.6)	9.9 (−207.8, 200.4)	143.4 (−91.3, 340.4)	−12.6 (−25.3, 1.4)	2.0 (−17.5, 24.7)	22.3 (−9.7, 62.1)
(c) Birth cohort						
Before 1945	−8.6 (−28.7, 7.8)	−6.9 (−35.5, 10.7)	−3.8 (−32.8, 12.2)	−22.5 (−61.0, 38.4)	−11.9 (−64.0, 81.9)	8.2 (−71.7, 189.7)
1945–1964	−68.2 (−174.3, 32.1)	68.9 (−64.9, 186.1)	157.2 (33.4, 261.4)	−10.0 (−23.7, 5.4)	13.2 (−9.4, 39.7)	48.4 (7.7, 99.6)
1965–1974	−100.3 (−173.3, −32.4)	−95.0 (−214.6, 7.4)	−65.3 (−208.2, 49.5)	−25.5 (−39.1, −9.7)	−20.7 (−39.5, 2.3)	−15.5 (−42.3, 19.5)
1975 or later	−29.4 (−113.4, 47.2)	22.2 (−128.4, 146.6)	86.2 (−112.7, 240.1)	−6.6 (−24.6, 14.4)	6.8 (−20.8, 40.8)	25.5 (−18.4, 84.5)
(d) Injection drug use status						
PWID	−196.0 (−324.0, −77.1)	−164.4 (−384.8, 28.1)	−14.5 (−295.1, 214.3)	−24.5 (−36.4, −11.0)	−16.7 (−34.1, 3.7)	0.5 (−27.3, 35.6)
Non-PWID	−12.3 (−126.1, 94.7)	119.8 (−20.1, 242.4)	188.1 (59.6, 296.7)	−1.3 (−16.2, 15.7)	21.7 (−2.7, 50.2)	55.4 (12.4, 109.2)

PWID = people who inject drugs, non-PWID = people who do not inject drugs.

## Data Availability

Aggregate data on the monthly number of individuals initiating hepatitis C treatment, including the data used for the interrupted time series analysis of our full study population, are reported in Table S4 (Supplementary Materials).

## Supplementary Materials

The following supporting information can be downloaded at: https://www.mdpi.com/article/10.3390/v16050655/s1, Definition of injection drug use; Table S1: data sources integrated within the BC COVID-19 Cohort (BCC19C); Table S2: prescription drugs used to define hepatitis C treatment initiation; Table S3: median monthly number of individuals initiating hepatitis C treatment during pre-policy period (April 2018–March 2020) in British Columbia, by population; Table S4: number of individuals initiating HCV treatment in British Columbia by month and year, 2018 to 2022; Figure S1: monthly number of individuals initiating hepatitis C treatment in British Columbia prior to COVID-19-related policies (April 2017 to March 2020); Figure S2: estimated number of individuals initiating HCV treatment following COVID-19-related policies with 95% confidence band, compared to counterfactual, stratified by sex.
