# Supplementary material for: Impact of the COVID-19 Pandemic on Hepatitis C Treatment Initiation in British Columbia, Canada: An Interrupted Time Series Study
Source: Viruses. 2024 Apr 23;16(5):655. doi: 10.3390/v16050655 (PMC11125629; doi:10.3390/v16050655)
Supplement: Supplementary file 1 [file viruses-16-00655-s001.zip › viruses-2971447-supplementary.pdf]

Supplementary Materials

Impact of the COVID-19 pandemic on hepatitis C treatment initiation in British Columbia, Canada: an interrupted time series study

Richard L. Morrow, Mawuena Binka, Julia Li, Mike Irvine, Sofia R. Bartlett, Stanley Wong, Dahn Jeong, Jean Damascene Makuza, Jason Wong, Amanda Yu, Mel Krajden, Naveed Zafar Janjua

Table of Contents

Definition of injection drug use 2

Table S1. Data Sources integrated within the BC COVID-19 Cohort (BCC19C) 3

Table S2. Prescription drugs used to define hepatitis C treatment initiation 4

Table S3. Median monthly number of individuals initiating hepatitis C treatment during pre-policy period (April 2018 - March 2020) in British Columbia, by population 5

Table S4. Number of individuals initiating HCV treatment in British Columbia by month and year, 2018 to 2022 6

Figure S1. Monthly number of individuals initiating hepatitis C treatment in British Columbia prior to COVID-19-related policies (April 2017 to March 2020) 7

Figure S2. Estimated number of individuals initiating HCV treatment following COVID-19-related policies with 95% confidence band, compared to counterfactual, stratified by sex 8

Supplementary references 9

Definition of injection drug use

Injection drug use was defined as the occurrence of at least 2 physician visits, 1 hospitalization, or 1 emergency department visit related to major drug-related diagnoses involving addiction, dependence, and drug-induced mental disorders; illicit drug use most likely to be injecting (e.g. excluding cannabis), or illicit use of prescribed drugs including: hallucinogens, barbiturates/tranquillizers, sedatives, hypnotics, anxiolytics, opioids, cocaine, amphetamine.

Diagnosis age was required to be between 11 and 65 years, inclusive.

Any individual who met this definition of injection drug use by the end of the study period (Dec 31, 2022) was classified as a person who injects drugs.

Physician billing data:

ICD-9 diagnostic codes: starting with 292, 970, 3040-42, 3044-49, 3054-57, 3059, 6483, 7960, 9621, 9650, 9658, 9663-64, 9670, 9684-85, 9694, 9696-99, 9700, 9701, 9708, 9709, E8500 ,or exact codes V6542, 9672, E9397

Hospital data:

ICD-9-CM (hospitalization data): starting with 292, 970, 3040-42, 3044-49, 3054-7, 3059, 6483, 7960, 9621, 9650, 9658, 9663-64, 9670, 9684-85, 9694, 9696-99, 9700, 9701, 9708, 9709, E8500 , or exact codes V6542, 9672, E9397.

ICD-10-CA (hospitalization or emergency department data): starting with F11, F13-5, F19, Z722 or exact codes R781-82, T387, T400-T406, T408-09, T412, T423-28, T436, T438-39, T507, T4020-23, T4028, T4040-41, T4048.

ICD-10 complaint codes (emergency department data): exact codes 751, 753.

Table S1. Data Sources integrated within the BC COVID-19 Cohort (BCC19C)

**British Columbia Centre for Disease Control (BCCDC), Provincial Heath Services Authority (PHSA) and Regional Health Authority data sources:**	**Data Date Ranges:**
Integrated COVID-19 laboratory dataset (SARS-CoV2 tests from private/public labs) [S1]	Jan,2020-onward
Integrated COVID-19 case surveillance data (positive laboratory tests and historical regional health authority case data integrated with vaccination, genomic screening/sequencing, hospitalization, and death data) [S2]	Jan,2020-onward
Provincial COVID-19 Monitoring Solution (critical and non-critical care hospital census data) [S3]	Jan,2020-onward
Provincial Immunizations Registry (COVID-19 vaccination data) [S4]	Dec,2020-onward
Provincial Laboratory Information Solution (laboratory tests from private/public labs) [S5]	Jan,2020-onward
Public Health Reporting Data warehouse (Influenza laboratory tests) [S6]	Jan,2008-onward
Emergency department visits (hospital-based and community-based ambulatory care)	Mar,2020-onward
**Ministry of Health (MoH) Administrative Data Sources:**	**Data Date Ranges:**
Client Roster (CR) (registry of enrollment in the universal public health insurance plan including residential history) [S7]	2008/9-onward
Discharge Abstracts Database (DAD) (hospital discharge records) [S8]	2008/9-onward
Medical Services Plan (MSP) (physician diagnostic and billing data for services provided through universal public health insurance plan) [S9]	2008/9-onward
PharmaNet (Pharma) (prescription drugs dispensed from community pharmacies, includes medications covered by public and private insurance plans) [S10]	2008/9-onward
BC Vital Statistics (VS) (deaths registry)[S11]	2008/9-onward
National Ambulatory Care Reporting System (NACRS) (hospital-based and community-based ambulatory care) [S12]	2011/12–onward
Chronic Disease Registry [S13]	2008/9-2018/19
811 Calls (respiratory calls only) [S14]	2014-onward
Health System Matrix [S15]	2018/19-onward
Population Grouper Methodology [S16]	2008/9-onward

Table S2. Prescription drugs used to define hepatitis C treatment initiation

Drug category	Generic name
a) Direct-acting antivirals	elbasvir/grazoprevir
	glecaprevir/pibrentasvir
	ledipasvir/sofosbuvir
	sofosbuvir
	sofosbuvir/velpatasvir
	sofosbuvir/velpatasvir/voxilaprevir
b) Other hepatitis C drugs	asunaprevir
	boceprevir
	daclatasvir
	ombitasvir/paritaprevir/ritonavir and dasabuvir
	peginterferon
	ribavirin
	ribavirin/peginterferon
	simeprevir
	telaprevir

Table S3. Median monthly number of individuals initiating hepatitis C treatment during pre-policy period (April 2018 - March 2020) in British Columbia, by population

Population	Number of individuals initiating hepatitis C treatment per month	
	Median	Interquartile range
a) Overall	256	89.5
b) Sex		
Female	90.5	34.25
Male	156	55
c) Birth cohort		
Before 1945	7.5	4.5
1945–1964	140.5	71
1965–1974	55	10.75
1975 or later	52	10.75
d) Injection drug use status		
People who inject drugs	143.5	72.2
People who do not inject drugs	109.5	24.75

Table S4. Number of individuals initiating HCV treatment in British Columbia by month and year, 2018 to 2022

Month	Number of individuals initiating HCV treatment by year				
	2018	2019	2020	2021	2022
January	248	296	218	155	105
February	223	276	211	127	119
March	282	277	192	150	91
April	316	266	138	157	113
May	403	244	137	142	115
June	393	246	157	146	135
July	348	219	137	118	109
August	322	196	105	100	126
September	271	195	150	117	112
October	338	215	157	104	93
November	298	213	126	117	129
December	213	169	125	72	95
Total, n	3,655	2,812	1,853	1,505	1,342
Difference from 2019, %	30.0	-	-34.1	-46.5	-52.3

(a) Overall

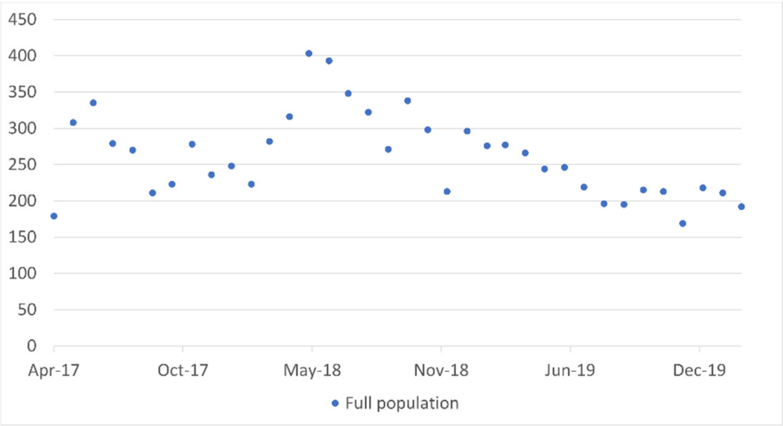

(b) Stratified by sex

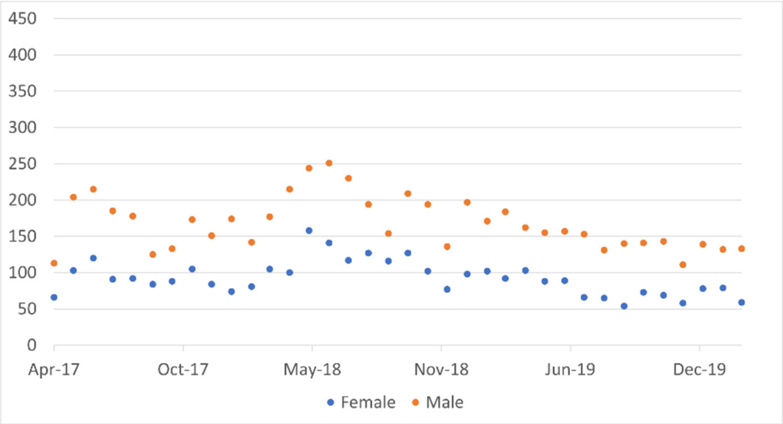

(c) Stratified by birth cohort

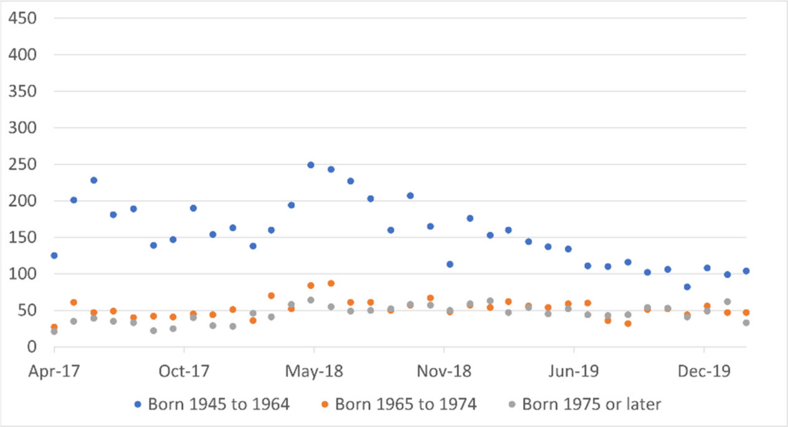

(d) Stratified by injection drug use status

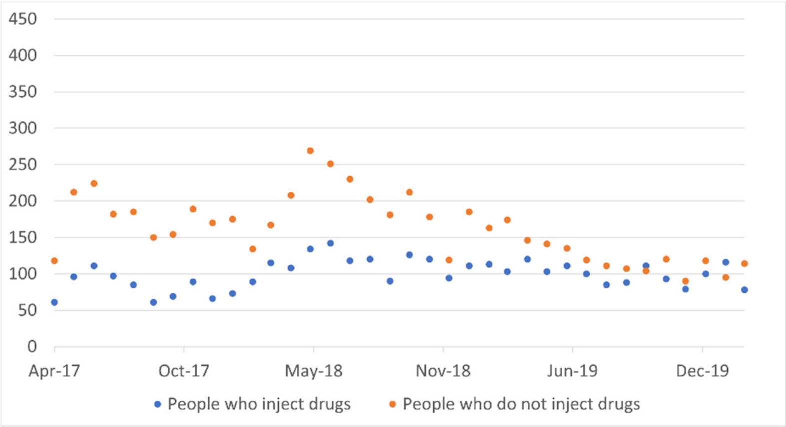

Figure S1. Monthly number of individuals initiating hepatitis C treatment in British Columbia prior to COVID-19-related policies (April 2017 to March 2020)

Figure S2. Estimated number of individuals initiating HCV treatment following COVID-19-related policies with 95% confidence band, compared to counterfactual, stratified by sex

(a) Female

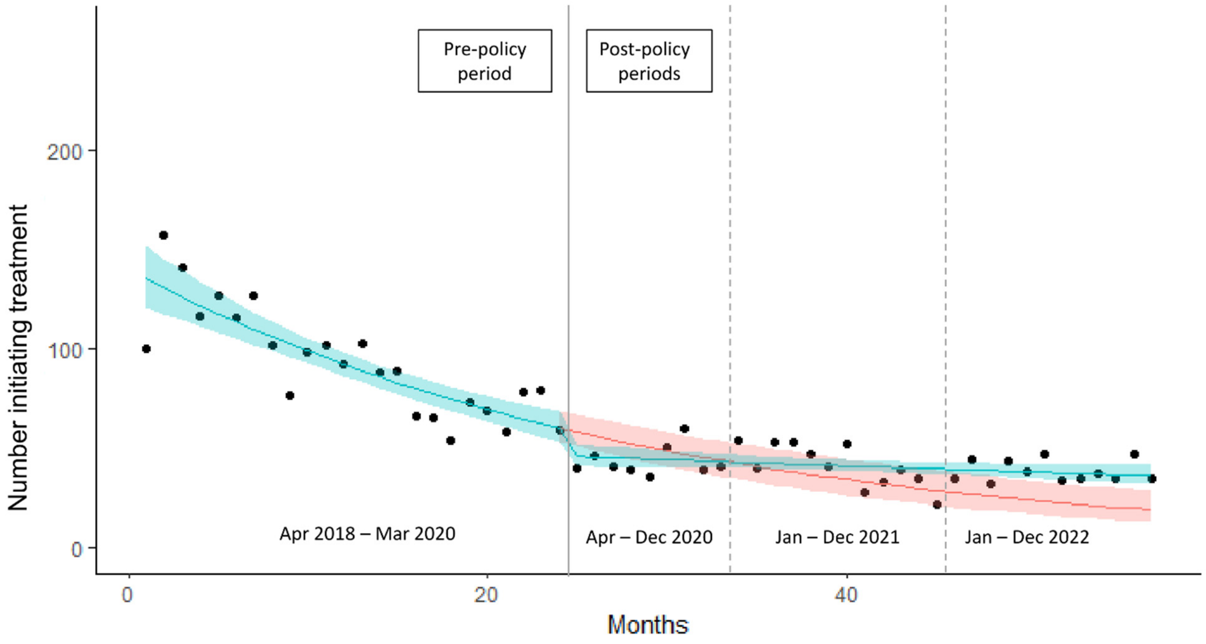

(b) Male

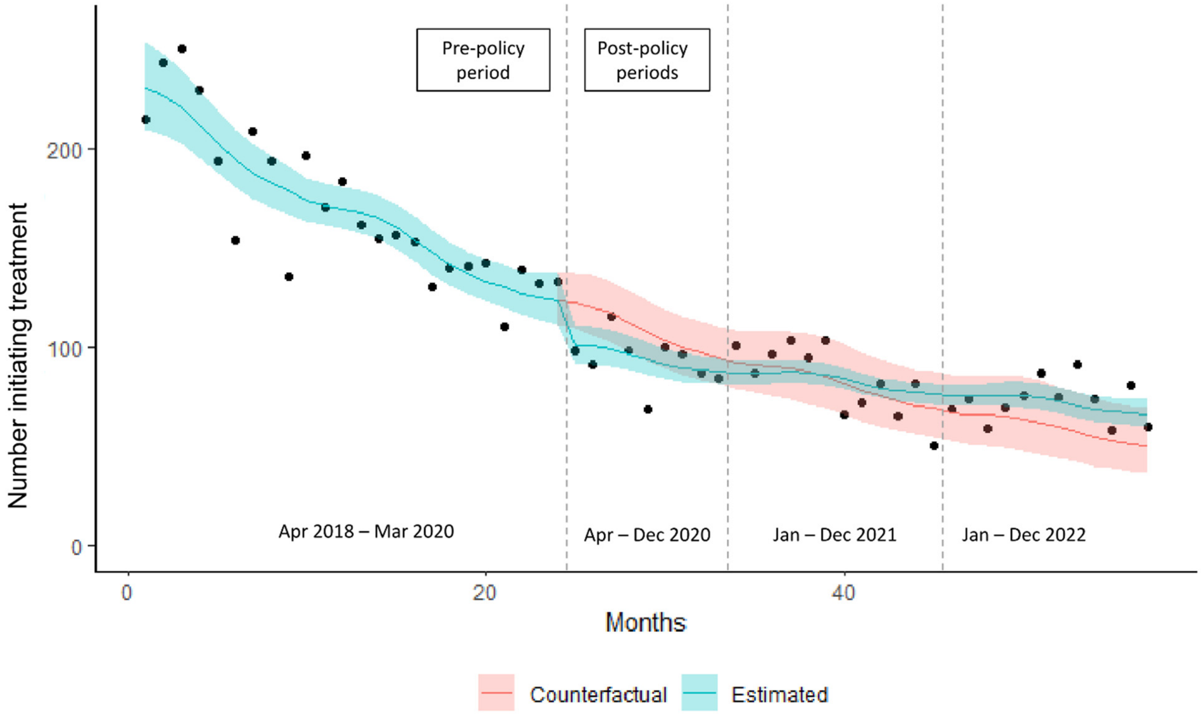

Supplementary references

S1. British Columbia Centre for Disease Control [creator]. Integrated COVID-19 laboratory dataset (SARS-CoV2 tests from private/public labs). Public Health Reporting Data Warehouse, British Columbia Centre for Disease Control [publisher] (2020). 2021.

S2. British Columbia Centre for Disease Control [creator]. Integrated COVID-19 case surveillance data. British Columbia Centre for Disease Control [publisher]. (2020). 2021.

S3. Provincial Health Services Authority [creator]. Provincial COVID-19 Monitoring Solution. Provincial Health Services Authority [publisher]. (2020). 2021.

S4. Provincial Health Services Authority [creator]. COVID-19 vaccination data. Provincial Immunizations Registry, Provincial Public Health Information Systems [publisher]. (2020). 2021.

S5. Provincial Health Services Authority [creator]. Provincial Laboratory Information Solution. Provincial Public Health Information Systems [publisher]. (2020). 2021.

S6. British Columbia Centre for Disease Control [creator]. Respiratory datamart, Public Health Reporting Data Warehouse, British Columbia Centre for Disease Control [publisher] (2020). 2021.

S7. British Columbia Ministry of Health [creator]. Client Roster (Client Registry System/Enterprise Master Patient Index). British Columbia Ministry of Health [publisher]. Data Extract. MOH (2020). 2021. https://www2.gov.bc.ca/gov/content/health/health-forms/online-services

S8. British Columbia Ministry of Health [creator]. Discharge Abstract Database (Hospital Separations). British Columbia Ministry of Health [publisher]. Data Extract. MOH (2020). 2021. https://www2.gov.bc.ca/gov/content/health/health-forms/online-services

S9. British Columbia Ministry of Health [creator]. Medical Services Plan (MSP) Payment Information File. British Columbia Ministry of Health [publisher]. Data Extract. MOH (2020). 2021.https://www2.gov.bc.ca/gov/content/health/health-forms/online-services

S10. British Columbia Ministry of Health [creator]. PharmaNet. British Columbia Ministry of Health [publisher]. Data Extract. MOH (2020). 2021. https://www2.gov.bc.ca/gov/content/health/health-forms/online-services

S11. BC Vital Statistics Agency [creator]. Vital Statistics Deaths. BC Vital Statistics Agency [publisher]. Data Extract. BC Vital Statistics Agency (2020). 2021. https://www2.gov.bc.ca/gov/content/health/health-forms/online-services

S12. British Columbia Ministry of Health [creator]. National Ambulatory Care Reporting System. British Columbia Ministry of Health [publisher]. Data Extract. MOH (2020). 2021. https://www2.gov.bc.ca/gov/content/health/health-forms/online-services

S13. British Columbia Ministry of Health [creator]. Chronic Disease Registry. British Columbia Ministry of Health [publisher]. Data Extract. MOH (2020). 2021. https://www2.gov.bc.ca/gov/content/health/health-forms/online-services

S14. British Columbia Ministry of Health [creator]. 811 calls. British Columbia Ministry of Health [publisher]. Data Extract. MOH (2020). 2021. https://www2.gov.bc.ca/gov/content/health/health-forms/online-services

S15. British Columbia Ministry of Health [creator]. Health System Matrix. British Columbia Ministry of Health [publisher]. Data Extract. MOH (2020). 2021. https://www2.gov.bc.ca/gov/content/health/health-forms/online-services

S16. British Columbia Ministry of Health [creator]. Population Grouper Methodology. British Columbia Ministry of Health [publisher]. Data Extract. MOH (2020). 2021. https://www2.gov.bc.ca/gov/content/health/health-forms/online-services
